# Supplementary material for: Synergistic Effect of a Defect-Free Graphene Nanostructure as an Anode Material for Lithium Ion Batteries
Source: Nanomaterials (Basel). 2019 Dec 18;10(1):9. doi: 10.3390/nano10010009 (PMC7023017; doi:10.3390/nano10010009)
Supplement: Supplementary file 1 [file nanomaterials-10-00009-s001.pdf]

Supplementary Materials

Synergistic Effect of a Defect-Free Graphene Nanostructure as an Anode Material for Lithium Ion Batteries

Kwang Hyun Park [1], Byung Gon Kim [2] and Sung Ho Song [3,*]

[1] Center for Artificial Low Dimensional Electronics System, Institute for Basic Science (IBS), Pohang-si, Gyeongsangbuk-do 37673, Korea; recite14@gmail.com

[2] Next Generation Battery Research Center, Korea Electrotechnology Research Institute, 12 Bulmosan-ro 10beon-gil, Seongsan-gu, Changwon-si, Gyeongsangnam-do 51543, Korea; byunggonkim@keri.re.kr

[3] Division of Advanced Materials Engineering, Kongju National University, Cheonan, Chungnam 32588, Korea

* Correspondence: shsong805@kongju.ac.kr; Tel.: +82-41-521-9379

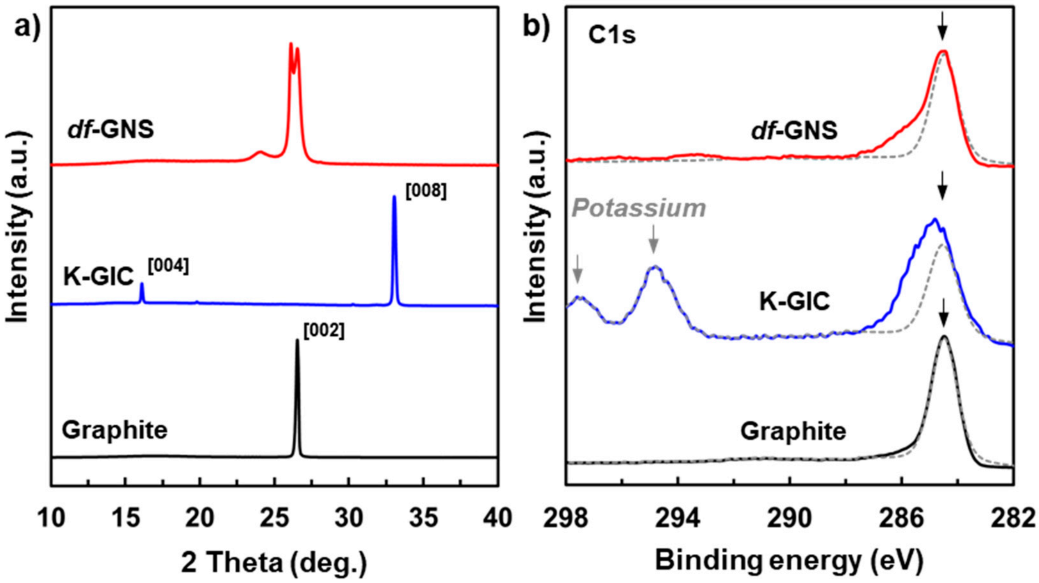

Figure S1. **a**, XRD patterns for the graphite, KC_8, and *df*-GNS. **b**, Chemical compositions of the graphite, KC_8, and *df*-GNS. (C1s peaks (C-C, 284.5 eV)).
